# SARS-CoV-2 seroprevalence and associated factors of infection before and after the Delta wave in French Polynesia: a cross-sectional study

**DOI:** 10.1186/s12889-024-17869-4

**Published:** 2024-02-05

**Authors:** Vincent Mendiboure, Iotefa Teiti, Maite Aubry, Anita Teissier, Tuterarii Paoaafaite, Jessica Vanhomwegen, Jean-Claude Manuguerra, Arnaud Fontanet, Van-Mai Cao-Lormeau, Yoann Madec

**Affiliations:** 1 Emerging Diseases Epidemiology Unit, Institut Pasteur, Université Paris Cité, 75015 Paris, France; 2 Laboratory of Research On Emerging Viral Diseases, Institut Louis Malardé, Papeete, Tahiti French Polynesia; 3 Environment and Infectious Risks Unit, Laboratory for Urgent Response to Biological Threats (CIBU), Institut Pasteur, Université Paris Cité, 75015 Paris, France; 4 Conservatoire National Des Arts Et Métiers, Unité PACRI, 75003 Paris, France

**Keywords:** SARS-CoV-2, COVID-19, Seroprevalence, Risk factor, General population, French Polynesia, Pacific islands

## Abstract

**Background:**

French Polynesia (FP) comprises 75 inhabited islands scattered across five archipelagos. Between July and October 2021, the SARS-CoV-2 Delta variant triggered a much stronger second epidemic wave in FP than the original Wuhan strain, which was dominant from August 2020 to March 2021. Although previous seroprevalence surveys made it possible to determine the proportion of the population infected by SARS-CoV-2 on the two most populated islands (Tahiti and Moorea) after the first (20.6% in Tahiti and 9.4% in Moorea) and second (57.7% in Tahiti) epidemic waves, no data are available for more remote islands. We used blood samples and personal data collected before, during, and after the second wave from inhabitants of several islands within the five archipelagos to assess the prevalence of SARS-CoV-2 infections and identify associated factors.

**Methods:**

Blood samples and personal data were collected between April and December 2021 as part of the MATAEA study, a cross-sectional survey conducted on a random sample of the adult population representative of the five FP archipelagos and stratified by age and gender. IgG antibodies targeting the SARS-CoV-2 nucleocapsid (N) protein were detected using a recombinant antigen-based microsphere immunoassay. Factors associated with anti-SARS-CoV-2-N seropositivity were identified using logistic regression models.

**Results:**

Of 1,120 participants, 503 (44.9%) tested positive for anti-SARS-CoV-2-N antibodies, corresponding to a weighted prevalence of 56.8% for the FP population aged 18–69 years. The seroprevalence increased from 21.9% to 62.1% before and during/after the Delta wave. Of these infections, only 28.4% had been diagnosed by health professionals. The odds of being seropositive were lower in males, participants recruited before the Delta wave, those who had never been married, those with a diagnosed respiratory allergy, smokers, and those vaccinated against COVID-19.

**Conclusions:**

Our results confirm the high impact of the Delta wave in FP. By the end of 2021, 56.8% of the FP population aged 18–69 years had been infected by SARS-CoV-2; the majority of these infections went undetected. Individuals with respiratory allergies were found to be less susceptible to SARS-CoV-2 infection.

**Supplementary Information:**

The online version contains supplementary material available at 10.1186/s12889-024-17869-4.

## Background

**Fig. 1:**
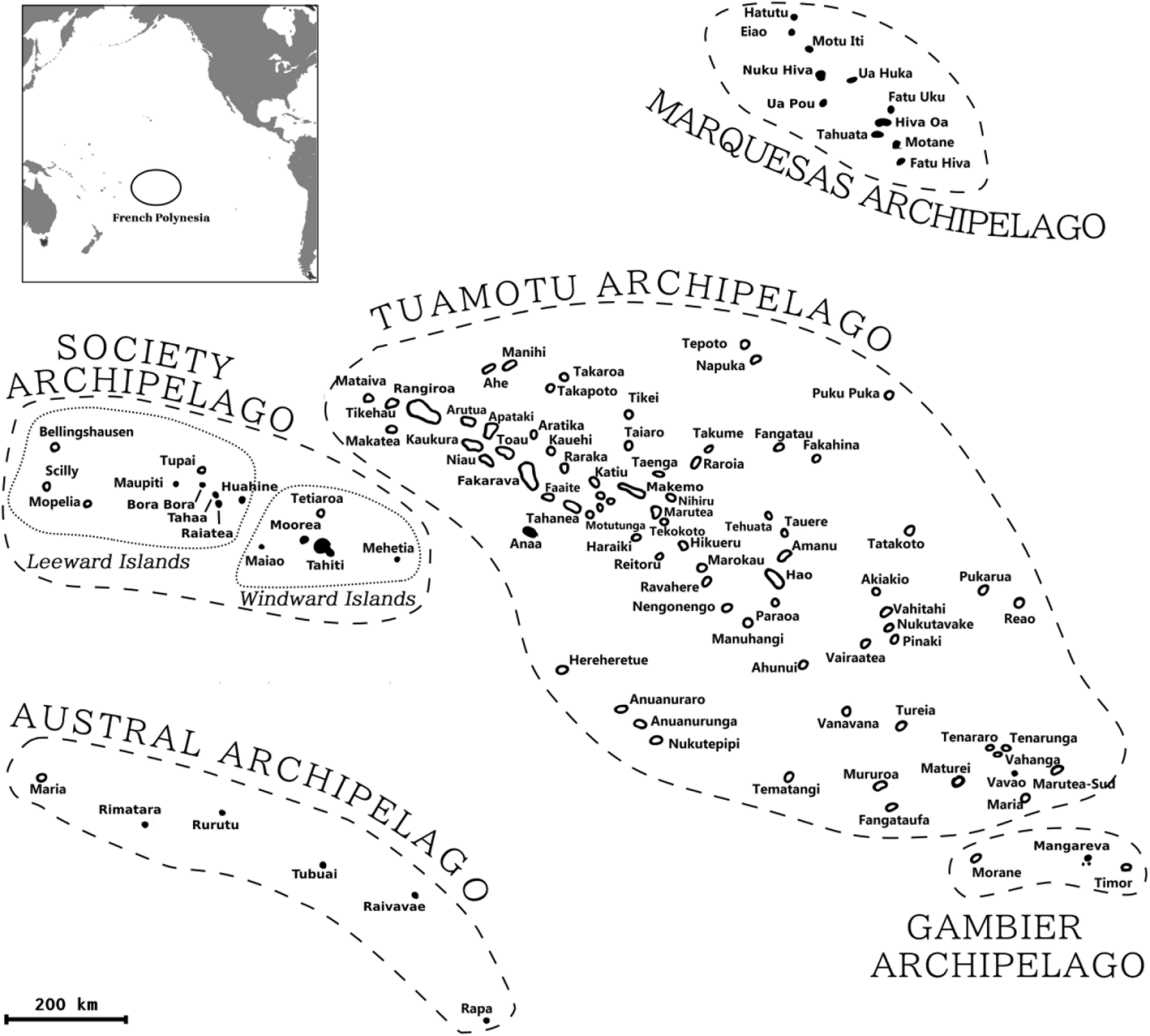
Map of French Polynesia. Each archipelago is delimited by dashed lines. The Windward and Leeward Islands are delimited by dotted lines*.* The inserted map in the upper left corner shows the location of French Polynesia (black circle) in the Pacific Ocean

French Polynesia (FP) is an overseas territory of the French Republic in the South Pacific. FP consists of 121 islands scattered over five million square kilometers [1] and grouped into five archipelagos (Fig. 1): the Society archipelago comprising the Windward Islands (WIs) and the Leeward Islands (LIs), as well as the Tuamotu, Gambier, Austral, and Marquesas archipelagos. In 2021, it was estimated that 279,550 people lived on 75 FP islands [2], with 75% of the population concentrated on the two islands of Tahiti and Moorea in the WIs [3].

Coronavirus disease 2019 (COVID-19), a highly contagious infection caused by the severe acute respiratory syndrome coronavirus 2 (SARS-CoV-2), was first identified in Wuhan, China, in December 2019 [4, 5] and declared a global pandemic by the World Health Organization (WHO) in March 2020 [6]. The first case of COVID-19 was detected in FP in March 2020 [7]. By the end of 2021, two epidemic waves had occurred in FP. The first wave, caused by the original Wuhan strain, started in August 2020 and peaked in October 2020, with nearly 18,000 cases confirmed over six months [8]. The second wave caused by the Delta variant hit FP more severely from July to September 2021, peaking in August 2021, and resulted in over 28,000 cases [9].

The vaccination campaign against COVID-19 started in FP in January 2021 using the Comirnaty® vaccine from Pfizer–BioNTech (USA/Germany). The Janssen® vaccine from Johnson & Johnson (USA) was introduced into the vaccination strategy from May 2021. By the end of 2021, 74% of the population aged 18 years and older had a complete vaccination schedule [10].

In February 2021, at the end of the first COVID-19 epidemic wave, a seroprevalence survey was performed on a random sample of 463 unvaccinated adults from Tahiti (*N* = 372) and Moorea (*N* = 91) [8]. The prevalence of anti-SARS-CoV-2 IgG antibodies was 20.6% [95% confidence interval (CI):16.3–25.6] in Tahiti and 9.4% [95% CI: 4.6–18.3] in Moorea (unpublished data). Another serosurvey was conducted during November–December 2021, i.e. after the second epidemic wave, from a random sample of 673 inhabitants of Tahiti aged 18 years and over [11]. Evidence of natural SARS-CoV-2 infection was found in 57.7% of the study’s participants.

Existing serological data only cover the WI population while epidemiological reports indicate that all the archipelagos of FP were affected during the first two COVID-19 epidemic waves [12]. To assess the prevalence of anti-SARS-CoV-2 antibodies and identify the factors associated with SARS-CoV-2 infection in the FP population before, during, and after the second epidemic wave, we used blood samples and related data collected from inhabitants of the five archipelagos between April and December 2021 as part of the MATAEA study, a cross-sectional survey designed to investigate health determinants in the FP adult population [13].

## Methods

### Study design

MATAEA is a cross-sectional survey conducted on a random sample of the general population aged 18–69 years and representative of the five archipelagos of FP, as previously published [13]. Briefly, the territory of FP was divided into three geographic strata: the WIs, the LIs, and the other archipelagos (i.e. Tuamotu, Austral, Gambier, and Marquesas), which respectively include 75.1%, 12.8%, and 12.0% of the population according to the 2017 census [3]. In each stratum, the most populated islands with a healthcare center and an airport with regular flight rotations were selected. Finally, in each island, households were randomly selected and one resident per household was randomly recruited. The number of participants to enroll was the same in the three strata (*N* = 700), proportional to the population size of the islands selected for the study, and equally divided into three age groups (18–29, 30–44, and 45–69), each including 50% males and 50% females. Participation in the study involved agreeing to answer a questionnaire (including questions on demographic characteristics, lifestyle habits, and medical history), undergo physical measurements (including height, weight, waist circumference, and arterial pressure), and provide biological samples (including blood) for laboratory analyses.

### Participant inclusion

Inclusion visits for the MATAEA study took place from November 2019 to December 2021. Because of the COVID-19 epidemic waves and restriction measures implemented to prevent the spread of SARS-CoV-2, inclusions were interrupted between March and May 2020, then between July 2020 and April 2021, and finally between August and September 2021. Upon resumption in April 2021, COVID-19 was added to the scope of the study and the questionnaire was expanded with questions specific to this disease [13]. Consequently, only participants recruited from April 2021 were considered in the subsequent analyses.

### Serological analysis

Blood samples from participants collected in 5 mL serum separation tubes (Becton Dickinson, USA) were centrifuged for 15 min at 3,500 rpm to retrieve serum. Immunoglobulin class G (IgG) antibodies targeting the SARS-CoV-2 nucleocapsid (N) protein were detected in the serum with the MAGPIX NxTAG® system (Luminex, USA) using a recombinant antigen-based microsphere immunoassay (MIA), as previously described [14]. Recombinant antigen-coated beads used for the MIA were provided by the Cellule d’Intervention Biologique d’Urgence (CIBU, Institut Pasteur, Paris). For each serum tested, the median fluorescence intensity value obtained was compared to a cut-off value to determine the participant’s immunological status (i.e. positive or negative for the detection of anti-SARS-CoV-2-N IgG antibodies). The cut-off value was calculated using receiver operating characteristic (ROC) analysis performed from a panel of positive and negative control sera.

### Definitions

Past SARS-CoV-2 infection was defined as a positive anti-N serology, given that the two COVID-19 vaccines used in FP between January and December 2021 do not elicit any antibody response to the SARS-CoV-2-N protein. In a sensitivity analysis, participants with a negative anti-N serology, but who reported having been tested positive for COVID-19 by a health professional before inclusion in the MATAEA study (i.e. who answered yes to the following question: have you had a nasopharyngeal test, performed by a nurse, that was positive for Covid-19?), were also considered as previously infected.

Information on COVID-19 vaccination status, as well as the type of vaccine used and the date of each injection if applicable, were collected from all participants in the questionnaire. Participants who self-reported having received at least one injection were considered as vaccinated. However, those who self-reported having been tested positive for COVID-19 by a health professional, prior to or within the same month as vaccination, were considered as unvaccinated for the analysis to reflect the time required for the vaccine to induce immune protection [15].

In another sensitivity analysis, participants were categorized as having been vaccinated for more or less than three months before inclusion to assess the impact of misclassifying the timing of infection compared to vaccination.

### Statistical analysis

In each archipelago, the prevalence of SARS-CoV-2 infections and associated 95% CI were estimated within the sample. Weighted prevalence was inferred from the prevalence in each stratum of the sample by weighting. Weights, defined by archipelago (except the Society archipelago, for which the WIs and LIs were treated separately, and the Tuamotu and Gambier archipelagos which were combined), were each participant’s inverse probability of selection, where the probability is the number of participants in each of the six age/sex strata divided by its total population.

Factors associated with anti-SARS-CoV-2 seroprevalence were identified using logistic regression models. Factors investigated were sociodemographic characteristics, medical history, behavior characteristics (physical activity and smoking habit), barrier gestures implemented, and vaccination status. First, a univariate analysis was performed. For the multivariate analysis, a stepwise forward procedure was applied. As the study was conducted sequentially on the different archipelagos between April and December 2021 [13] (Austral and Tuamotu: April–May, Gambier: May, Marquesas and LIs: May–June, WIs: July then October to December 21), while the second wave of COVID-19 started in mid-July and peaked in mid-August 2021, the month of inclusion showed a strong confusion effect with several factors. This motivated the choice of a stepwise forward procedure, starting from a model that included only the month of inclusion. At each step of the forward procedure, all factors were investigated without restriction, and the most significant one was added to the model. The procedure stopped when all additional factors investigated presented a *p*-value greater than 0.05.

All statistical analyses were performed with Stata 17 software (StataCorp., College Station, TX, USA). Statistical significance was set at *p* < 0.05.

## Results

### Participant and population characteristics

Between April and December 2021, a total of 1,148 participants were enrolled in the MATAEA survey, including 1,120 (97.6%) for whom serological analysis results for the detection of anti-SARS-CoV-2-N antibodies were available. The sociodemographic characteristics of the 1,120 participants considered in the subsequent analyses are detailed in Supplementary Table 1. Most of the participants were recruited in the WIs (57.2%), followed by the Marquesas (19.4%), the LIs (9.2%), the Tuamotu and the Austral (5.3%), and the Gambier (3.7%). The proportion of men and mean age of the participants did not differ between archipelagos (*p* = 0.28 and *p* = 0.32, respectively), while a significant difference was found for the level of education (*p* < 0.001).

### Anti-SARS-CoV-2-N seroprevalence in study participants

**Fig. 2:**
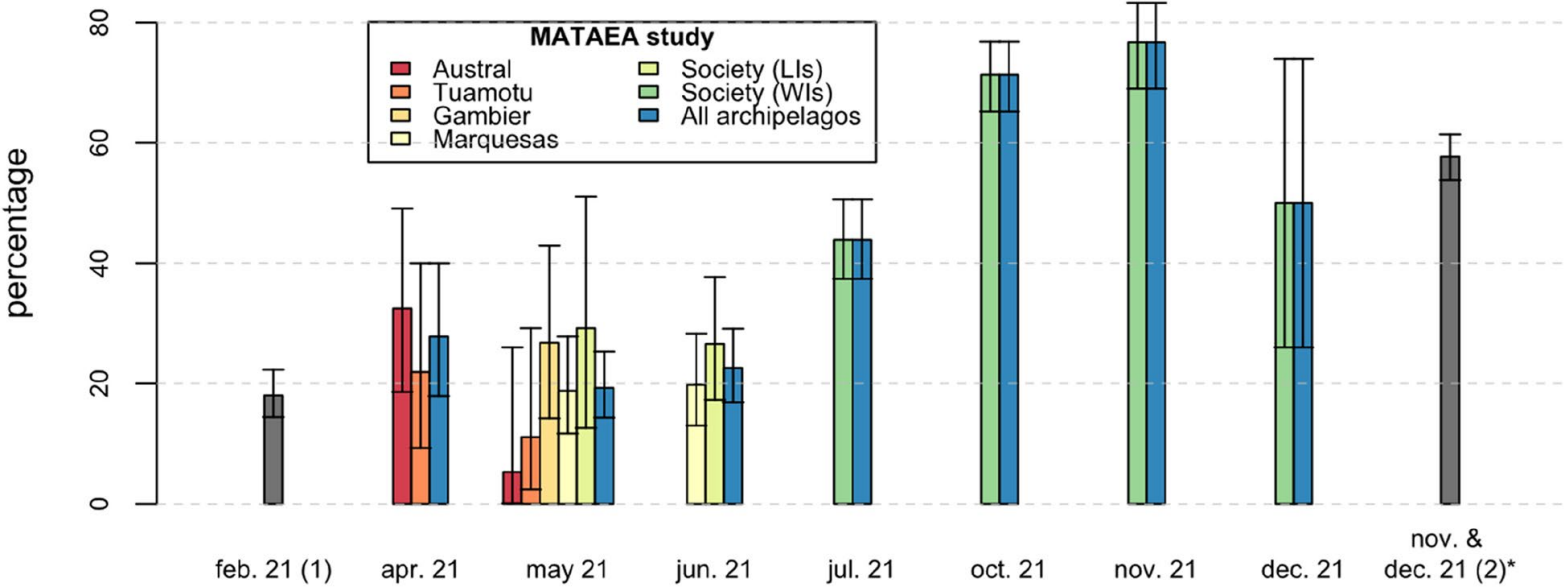
Proportion of participants with SARS-CoV-2 infection in MATAEA and two other serosurveys. LIs: Leeward Islands; WIs: Windward Islands. (1) Seroprevalence survey conducted in Tahiti and Moorea in February 2021^8^. (2) Seroprevalence survey conducted in Tahiti during November–December 2021^11^. *Confidence intervals were calculated based on the hypothesis of binomial distribution

Anti-SARS-CoV-2-N IgG antibodies, indicating past natural infection, were detected in 503/1,120 participants (44.9% [95% CI: 42.0–47.9]), corresponding to a weighted seroprevalence of 56.8% [95% CI: 53.4–60.2] for the FP population aged 18–69 years (Table 1). Before the Delta wave, from April to June 2021, anti-SARS-CoV-2-N antibodies were detected in 105/479 participants (21.9% [95% CI: 18.3–25.9]), versus 398/641 participants (62.1% [95% CI: 58.2–65.9]) during and after the Delta wave from July to December 2021. The proportions of participants with evidence of past infection significantly differed between age groups (*p* = 0.03), archipelagos (*p* < 0.001), and months of inclusion (*p* < 0.001), with the highest values found in individuals aged 18–29 years (50.1% [95% CI: 44.7–55.6]), those living in the WIs (62.1% [95% CI: 58.2–65.9]), and those enrolled in November 2021 (76.8% [95% CI: 69.0–83.3]) (Fig. 2).

**Table 1:** Prevalence of anti-SARS-CoV-2-N IgG antibodies among study participants

	**Participants n**	**Raw prevalence**			**Weighted prevalence**
		**n**	**% [95% CI]**	***p*-value**	**% [95% CI]**
**Total**	1,120	503	**44.9 [42.0–47.9]**		**56.8[53.4–60.2]**
**Age range (in years)**				0.03	
18–29	337	169	50.1 [44.7–55.6]		
30–44	408	184	45.1 [40.2–50.1]		
45–69	375	150	40.0 [35.0–45.2]		
**Gender**				0.57	
Male	536	236	44.0 [39.8–48.3]		
Female	584	267	45.7 [41.6–49.9]		
**Archipelago**				< 0.001	
Society (WIs)	641	398	62.1 [58.2–65.9]		61.4 [57.5–65.1]
Society (LIs)	103	28	27.2 [18.9–36.8]		26.8 [19.1–36.2]
Austral	59	14	23.7 [13.6–36.6]		24.5 [14.9–37.5]
Marquesas	217	42	19.4 [14.3–25.2]		19.0 [14.2–24.8]
Tuamotu	59	10	16.9 [8.4–29.0]		15.6 [8.5–26.9]
Gambier	41	11	26.8 [14.2–42.9]		25.9 [14.8–41.4]
**Period of inclusion**				< 0.001	
Before Delta wave (April-June 2021)	479	105	21.9 [18.3–25.9]		21.8 [18.1–25.9]
*April-21*	*72*	*20*	*27.8 [17.9–40.0]*		
*May-21*	*212*	*41*	*19.3 [14.3–25.3]*		
*June-21*	*195*	*44*	*22.6 [16.9–29.1]*		
During/after Delta wave (July-December 2021)	641	398	62.1 [58.2–65.9]		61.4 [57.5–65.1]
*July-21*	*230*	*101*	*43.9 [37.4–50.6]*		
*Octobre-21*	*247*	*176*	*71.3 [65.2–76.8]*		
*November-21*	*146*	*112*	*76.8 [69.0–83.3]*		
*December-21*	*18*	*9*	*50.0 [26.0–74.0]*		
**Self-reported number of SARS-CoV-2 infections**^a^				< 0.001	
0	967	360	37.2 [34.2–40.4]		
≥ 1	153	143	93.5 [88.3–96.8]		

*WIs* Windward Islands, *LIs* Leeward Islands

^a^Number of positive tests (RT-PCR or antigenic) for SARS-CoV-2 detection performed by a health professional, as self-reported by the participant

Moreover, the prevalence of anti-SARS-CoV-2-N antibodies was significantly higher in participants who self-reported at least one past SARS-CoV-2 infection than in those who did not (143/153 (93.5%) and 360/967 (37.2%), respectively; *p* < 0.001) (Table 1).

Participants were recruited in the five archipelagos at different periods: in the Tuamotu (Fakarava and Hao) and the Austral (Raivavae and Rimatara) during April–May 2021, the Gambier (Mangareva) in May, the Marquesas (Nuku Hiva, Hiva Oa, and Ua Pou) and the LIs (Huahine and Bora Bora) during May–June, and the WIs (Tahiti and Moorea) in July and then during October–December 2021 (Table 2 and Fig. 1). Due to collinearity between the month of inclusion and archipelago, related to occurrence of the Delta epidemic wave from July 2021, we only considered month of inclusion from these two variables for further analyses.

**Table 2:** Anti-SARS-CoV-2-N seroprevalence by month of inclusion and archipelago

	**April 2021**	**May 2021**	**June 2021**	**July 2021**	**October 2021**	**November 2021**	**December 2021**
**Archipelago**							
Austral	13/40 (32.5%)	1/19 (5.3%)					
Tuamotu	7/32 (21.9%)	3/27 (11.1%)					
Gambier		11/41 (26.8%)					
Marquesas		19/101 (18.8%)	23/116 (19.8%)				
Society (LIs)		7/24 (29.2%)	21/79 (26.6%)				
Society (WIs)				101/230 (43.9%)	176/243 (71.3%)	112/146 (76.7%)	9/18 (50.0%)
***p*-value**	0.32	0.16	0.27	**-**	**-**	**-**	**-**

*WIs *Windward Islands, *LIs *Leeward Islands

### Factors associated with SARS-CoV-2 infection

In the multivariate analysis, positivity for the detection of anti-SARS-CoV-2-N antibodies was significantly associated with the month of inclusion (*p* < 0.001). Indeed, participants recruited during the months of October and November 2021 were more likely to be positive than those recruited in July 2021, while participants recruited in April, May, and June were less likely to be positive (Table 3). Male participants were significantly less likely to be seropositive (*p* = 0.02). Regarding marital status, participants who had never been married (and were single at the time of inclusion) were significantly less likely to have been infected than those in a relationship (married or cohabiting) (*p* = 0.01). Finally, the prevalence of anti-N antibodies was significantly lower in participants with physician-diagnosed respiratory allergy (*p* = 0.03), those who smoked tobacco products (*p* = 0.04), and those who had been vaccinated (*p* < 0.001). In a sensitivity analysis, those vaccinated within the three months preceding inclusion, and those vaccinated more than three months before inclusion, were distinguished. Again, infection rate was significantly lower in the vaccinated, and the effect was stronger in those vaccinated more than three months before inclusion than in those vaccinated within three months of inclusion (*p* = 0.019). The effect of the other factors remained the same. In another sensitivity analysis where the ten participants with a negative anti-N serology, but who self-reported having been tested positive for COVID-19 by a health professional before inclusion, were also considered as previously infected; the same variables as above were found to be associated with prior infection, with similar odds ratios (data not shown).

**Table 3:** Analysis of factors associated with SARS-CoV-2 infection in the study participants (logistic regression)

	**Participants**	**Anti-N positive**	**OR [95% CI]**	***p*-value**^**(1)**^	**aOR [95% IC]**	***p*-value**^**(2)**^	
	**n**	**n (%)**					
**Total**	**1,120**	**503 (44.9)**					
**Month of inclusion**				< 0.001		< 0.001	
April	72	20 (27.8)	0.49 [0.28–0.88]		0.49 [0.26–0.93]		
May	212	41 (19.3)	0.31 [0.20–0.47]		0.35 [0.23–0.55]		
June	195	44 (22.6)	0.37 [0.24–0.57]		0.42 [0.27–0.65]		
July	230	101 (43.9)	1 (Ref.)		1 (Ref.)		
October	247	176 (71.3)	3.17 [2.17–4.63]		4.87 [3.21- 7.37]		
November	146	112 (76.7)	4.21 [2.65–6.69]		7.17 [4.28–12.00]		
December	18	9 (50.0)	1.28 [0.49–3.34]		2.56 [0.90–7.23]		
**Archipelago**				< 0.001			
Society (WIs)	641	398 (62.1)	1 (Ref.)				
Society (LIs)	103	28 (27.2)	0.23 [0.14–0.36]				
Austral	59	14 (23.7)	0.19 [0.10–0.35]				
Marquesas	217	42 (19.4)	0.15 [0.10–0.21]				
Tuamotu	59	10 (17.0)	0.12 [0.06–0.25]				
Gambier	41	11 (26.8)	0.22 [0.11–0.45]				
**Island**				< 0.001			
***Society (WIs)***							
Tahiti	593	367 (61.9)	1 (Ref.)				
Moorea	48	31 (64.6)	1.12 [0.61–2.08]				
***Society (LIs)***							
Bora Bora	42	16 (38.1)	0.38 [0.20–0.72]				
Huahine	61	12 (19.7)	0.15 [0.08–0.29]				
***Austral***							
Rimatara	19	1 (5.3)	0.03 [0.00–0.26]				
Raivavae	40	13 (32.5)	0.30 [0.15–0.59]				
***Marquesas***							
Nuku Hiva	101	19 (18.8)	0.14 [0.08–0.24]				
Hiva Oa	63	9 (14.3)	0.10 [0.05–0.21]				
Ua Pou	53	14 (26.4)	0.22 [0.12–0.42]				
***Tuamotu***							
Fakarava	32	7 (21.9)	0.17 [0.07–0.41]				
Hao	27	3 (11.1)	0.08 [0.02–0.26]				
***Gambier***							
Mangareva	41	11 (26.8)	0.23 [0.11–0.46]				
**INSEE classification of municipalities**				< 0.001			
Rural area	709	278 (39.2)	0.53 [0.42–0.68]				
Urban area	411	225 (54.7)	1 (Ref.)				
**Gender**				0.57		0.02	
Male	536	236 (44.0)	0.93 [0.74–1.18]		0.71 [0.53–0.95]		
Female	584	267 (45.7)	1 (Ref.)		1 (Ref.)		
**Age range (in years)**				0.02			
18–29	337	169 (50.1)	1.22 [0.92–1.64]				
30–44	408	184 (45.1)	1 (Ref.)				
45–69	375	150 (40.0)	0.81 [0.61–1.08]				
**Socio-cultural background**				0.41			
Polynesian	841	385 (45.8)	1 (Ref.)				
Caucasian	53	19 (35.8)	0.66 [0.37–1.18]				
Asian	10	2 (20.0)	0.30 [0.06–1.40]				
Mixed race	200	90 (45.0)	0.97 [0.71–1.32]				
Other	8	4 (50.0)	1.18 [0.29–4.77]				
**Level of education**				0.94			
Primary school (or before)	164	70 (42.7)	0.92 [0.64–1.32]				
Secondary school	276	126 (45.7)	1.03 [0.76–1.40]				
High school (or equivalent)	433	194 (44.8)	1 (Ref.)				
University or after	246	113 (45.9)	1.05 [0.76–1.43]				
Missing data	1	0 (0.0)	0.41 [0.02–10.13]				
**Marital status**^a^				0.05		0.01	
Never married	292	112 (38.4)	0.71 [0.54–0.94]		0.64 [0.46–0.89]		
In a relationship	756	353 (46.7)	1 (Ref.)		1 (Ref.)		
Separated, divorced, widowed	70	37 (52.9)	1.28 [0.78–2.09]		1.69 [0.96–2.97]		
Missing data	2	1 (50.0)	1.14 [0.07–18.32]		0.61 [0.03–11.92]		
**Professional occupation**				< 0.001			
Government employee	173	63 (36.4)	0.52 [0.35–0.76]				
Private employee (and volunteers)	272	143 (52.6)	1 (Ref.)				
Self-employed	184	62 (33.7)	0.46 [0.31–0.68]				
Student	40	21 (52.5)	1.00 [0.51–1.94]				
No outside activity	451	214 (47.5)	0.81 [0.60–1.10]				
**Number of people in the household**				< 0.001			
1–2	229	80 (34.9)	0.63 [0.46–0.86]				
3–5	609	281 (46.1)	1 (Ref.)				
6–10	248	119 (48.0)	1.08 [0.80–1.45]				
≥ 11	34	23 (67.6)	2.44 [1.17–5.09]				
**Body Mass Index (kg/m**^**2**^**)**				0.60			
< 25	222	95 (42.8)	1 (Ref.)				
25–29	290	128 (44.1)	1.06 [0.74–1.50]				
≥ 30	598	277 (46.3)	1.15 [0.85–1.57]				
Missing data	10	3 (30.0)	0.57 [0.14–2.27]				
**High blood pressure**^b^				0.05			
No	714	340 (47.6)	1 (Ref.)				
Yes	405	163 (40.2)	0.74 [0.58–0.95]				
Missing data	1	0 (0.0)	0.37 [0.01–9.03]				
**Diabetes**^c^				0.31			
No	1018	464 (45.6)	1 (Ref.)				
Yes	91	34 (37.4)	0.71 [0.46–1.11]				
Missing data	11	5 (45.5)	0.99 [0.30–3.28]				
**Respiratory allergy**^d^				0.47			0.03
Yes	99	41 (41.4)	0.86 [0.57–1.31]		0.54 [0.33–0.89]		
No	1013	457 (45.1)	1 (Ref.)		1 (Ref.)		
Missing data	8	5 (62.5)	2.03 [0.48–8.53]		2.46 [0.45–13.60]		
**Asthma**^d^				0.35			
Yes	212	99 (46.7)	1.08 [0.80–1.46]				
No	904	404 (44.7)	1 (Ref.)				
Missing data	4	0 (0.0)	0.14 [0.01–2.56]				
**Long-term illness**				0.62			
Yes	176	76 (43.2)	0.92 [0.67–1.27]				
No	944	427 (45.2)	1 (Ref.)				
**History of cancer**				0.25			
Yes	32	11 (34.4)	0.64 [0.31–1.33]				
No	1085	492 (45.3)	1 (Ref.)				
Missing data	3	0 (0.0)	0.17 [0.01–3.34]				
**Level of physical activity**^e^				0.95			
Limited	347	160 (46.1)	1.05 [0.75–1.46]				
Moderate	240	108 (45.0)	1 (Ref.)				
High	531	234 (44.1)	0.96 [0.71–1.31]				
Missing data	2	1 (50.0)	1.22 [0.08–19.77]				
**Smoking**				0.03		0.04	
Yes	455	187 (41.1)	0.77 [0.61–0.98]		0.74 [0.56–0.98]		
No	665	316 (47.5)	1 (Ref.)		1 (Ref.)		
**Preventive measure: Hand hygiene**				0.06			
No	135	48 (35.6)	1 (Ref.)				
Yes	984	455 (46.2)	1.55 [1.07–2.25]				
Missing data	1	0 (0.0)	0.60 [0.02–15.05]				
**Preventive measure: Not shaking hands**				0.07			
No	350	140 (40.0)	1 (Ref.)				
Yes	769	363 (47.2)	1.34 [1.04–1.73]				
Missing data	1	0 (0.0)	0.50 [0.02–12.35]				
**Preventive measure: Coughing into the elbow**				0.01			
No	494	197 (39.9)	1 (Ref.)				
Yes	625	306 (49.0)	1.45 [1.14–1.83]				
Missing data	1	0 (0.0)	0.50 [0.02–12.39]				
**Preventive measure: Physical distancing**				< 0.001			
No	309	111 (35.9)	1 (Ref.)				
Yes	810	392 (48.4)	1.67 [1.27–2.19]				
Missing data	1	0 (0.0)	0.59 [0.02–14.69]				
**Preventive measure: Wearing mask**				0.83			
No	83	36 (43.4)	1 (Ref.)				
Yes	1036	467 (45.1)	1.07 [0.68–1.67]				
Missing data	1	0 (0.0)	0.43 [0.02–10.96]				
**Vaccination status before SARS-CoV-2 infection (if any)**				< 0.001		< 0.001	
Not vaccinated	645	325 (50.4)	1 (Ref.)		1 (Ref.)		
Vaccinated	441	170 (38.5)	0.62 [0.48–0.79]		0.38 [0.28–0.52]		
Missing data	34	8 (23.5)	0.30 [0.14–0.68]		0.82 [0.33–2.06]		

*OR* Odds ratio, *aOR* Adjusted odds ratio, *CI* Confidence interval, *INSEE* Institut National de la Statistique et des Etudes Economiques (https://www.insee.fr/fr/statistiques/3651609)

(1) Likelihood-ratio test

(2) Wald test

^a^'Never married' excludes cohabiting participants and ‘In a relationship’ includes married and cohabiting participants

^b^High blood pressure was defined as systolic blood pressure of 140 mmHg or higher, or diastolic blood pressure of 90 mmHg or higher, or taking blood pressure medication in the last two weeks (self-reported)

^c^Diabetes was defined as a glycated hemoglobin of 6.5% or above or taking antidiabetic medication in the last two weeks (self-reported)

^d^Respiratory allergy and asthma were declarative variables in response to the respective questions "Has a doctor or other health professional ever told you that you have known respiratory allergies (dust, pollen, animals)?" and "Has a doctor or other health professional ever told you that you have asthma?"

^e^Level of physical activity was defined according to WHO criteria

The following variables were significantly associated with the prevalence of anti-N antibodies in the univariate analysis but were no longer associated after adjustment: INSEE classification of municipality (urban/rural, *p* < 0.001 in univariate), age group (*p* = 0.02), professional occupation (*p* < 0.001), number of people living in the household (*p* < 0.001), coughing into the elbow as a protective measure (*p* = 0.01), and social distancing as a protective measure (*p* < 0.001).

## Discussion

This serosurvey, conducted between April and December 2021 on a sample of 1,120 adults from the five archipelagos of FP, investigated the seroprevalence of SARS-CoV-2 and factors associated with infection before, during, and after the second COVID-19 epidemic wave caused by the Delta variant. During the whole study period, 44.9% of the participants had detectable anti-SARS-CoV-2-N IgG antibodies indicating previous natural infection, which represented 56.8% [95% CI: 53.4–60.2] of the population aged 18 to 69 years. During the period from April to June (i.e. before the Delta wave), the presence of anti-N antibodies was detected in 21.9% (95% CI: 18.3–25.9) of the participants included in the LIs, Austral, Marquesas, and Tuamotu and Gambier. This result is consistent with the overall SARS-CoV-2 seroprevalence of 18% found in a previous serosurvey conducted in February 2021 in the WIs (20.6% [95% CI: 16.3–25.6] in Tahiti and 9.4% [95% CI: 4.6–18.3] in Moorea) (unpublished data). Then, during the period from July to December 2021, which encompasses the Delta wave, positive anti-N serology was found in 62.1% [95% CI: 58.2–65.9] of the participants in the WIs. This seroprevalence, almost three times higher than before the Delta wave began, is consistent with both (i) epidemiological data recorded in FP, with a more than twofold increase in the cumulated number of COVID-19 cases between the end of June and mid-December 2021 [9, 16], and (ii) the results of another serosurvey conducted in Tahiti after the Delta wave ended, with anti-SARS-CoV-2-N IgG antibodies detected in 57.7% of the participants [11].

Interestingly, among the 153 participants who reported previous positive tests for COVID-19, 10 (6.5%, 95% CI: 3.2–11.7) no longer presented detectable anti-SARS-CoV-2-N antibodies. This result suggests a disappearance, or decrease below the serological assay detection limit, of IgG antibodies in previously infected participants, consistent with earlier publications [17–19]. Conversely, among the 503 participants with anti-N antibodies, 360 (71.6%, 95% CI: 67.4–75.5) had not previously been tested positive for COVID-19 by a health professional. This suggests a high rate of undiagnosed infections and an underestimation of COVID-19 cases in the surveillance data. The reasons for this discrepancy are likely multifactorial. The relatively high proportion of asymptomatic infections described in the literature indicates that they may account for some of the undetected cases [20, 21]. The use of self-tests available in pharmacies since August 2021 [22], reluctance to be screened, and limitations in contact tracing and screening programs may also contribute to the number of unreported cases. These aspects were not investigated in this study, making it impossible to measure their impact.

As expected, a significant association between the prevalence of anti-N antibodies and month of inclusion was evidenced in the multivariate analysis (*p* < 0.001). This association reflects the above-mentioned COVID-19 epidemiological dynamics in FP. Before the second wave (i.e. early July 2021), FP reported 19,000 cumulated COVID-19 cases, or 6.8% of the total population [16]. In the following three months, the Delta wave was responsible for 28,000 new infections, or 10% of the population [9].

In the multivariate analysis, men were found to be at lower risk of SARS-CoV-2 infection than women (*p* = 0.02). While the association between infection and gender has already been frequently identified, males were generally found to be at greater risk [23]. Nevertheless, some studies carried out in the general population have results consistent with ours, i.e. men are less at risk than women [24]. Our study does not provide a further explanation for this association.

Our analyses also showed that single participants who had never been married were at a lower risk of infection than participants currently in relationships, whether married or cohabiting (*p* = 0.01), while age and number of people living in the household were not associated with infection. The importance of transmission within the couple among the chains of infection could explain this association. Interestingly, separated, divorced, or widowed participants did not have a significantly different infection risk than married or cohabiting participants.

We also found a significantly lower prevalence of anti-N antibodies in participants with respiratory allergy (*p* = 0.03). This could be explained by the protective measures taken individually and/or collectively by these participants and their entourage because of the perception of an increased risk of severity related to their condition [25–28]. Additionally, it has been suggested that the presence of respiratory allergy may be a protective factor against infection due to reduced expression of the SARS-CoV-2 receptor ACE2 in the nasal and respiratory epithelium [29, 30], and the protection of allergic rhinitis against COVID-19 has been confirmed in an epidemiological study [31]. However, our study did not provide evidence of an association between asthma and SARS-CoV-2 infection.

When analyzing the participants’ smoking status, we found a significantly lower risk of infection in those who smoked (*p* = 0.04). While several studies have reported similar results [32–35], the association and possible casual pathway are still unclear and under discussion. To counterbalance this message, it must be noted that smoking has been found to be associated with increased severity in cases of SARS-CoV-2 infection [36, 37].

Finally, we detected a decreased risk of infection in vaccinated participants (*p* < 0.001), consistent with previous reports [15, 38, 39]. However, and typically in cross-sectional studies, it was difficult to quantify the effect of vaccination because the sequence between infection and vaccination was unknown for many participants. Indeed, the majority of those found to have been infected by SARS-CoV-2 did not report having tested positive for COVID-19. In our analysis, vaccinated participants with evidence of past infection were systematically considered to have been infected after being vaccinated. This led to an underestimation of the effect of vaccination. On the other hand, among participants with no evidence of past infection, those who reported being vaccinated were systematically considered as such. This led to an overestimation of the effect of vaccination. Therefore, estimating the real effect of vaccination is complex. In an attempt to capture the effect of vaccination duration, we distinguished those vaccinated within three months preceding inclusion from those vaccinated more than three months prior to inclusion. Once again, vaccination was associated with lower odds of infection, and even more so among those who were vaccinated more than three months prior to inclusion.

This study suffers from some limitations. First, participants were included sequentially across FP concurrent with the strong time dynamic of the epidemics. Consequently, we could not adjust the analysis to investigate a possible association with the infection risk on the archipelagos. Another limitation relates to the fact that most of the variables were self-reported, so standard classification biases, memory bias, and social desirability bias could be expected. However, the questions on COVID-19 were asked in the middle of the pandemic. As a result, it can be considered that the answers were based on relatively recent events in participants’ memories, particularly when stating whether or not they had tested positive, thereby limiting bias. These biases, which were mostly non-differential, probably had little impact on our analysis. Moreover, all interviewers underwent training prior to the study’s implementation to ensure a consistent approach to the formulation of questions and data collection. Finally, this cross-sectional study did not allow us to investigate multiple SARS-CoV-2 infections in the participants.

## Conclusions

To our knowledge, this is the first SARS-CoV-2 seroprevalence study to cover the entire territory of FP and to analyze the determinants of the infection. While the Omicron variant had not yet reached FP, our study shows that 57% of the FP population aged 18 to 69 years had already been infected by SARS-CoV-2 by December 2021. One important finding was the high rate of undiagnosed infections. This highlights the need for wider diagnosis and screening access for future epidemic responses.

### Supplementary Information

**Additional file 1: Supplementary Table 1.** Sociodemographic characteristics of the study participants with available serological results for the detection of anti-SARS-CoV-2-N antibodies.

## Data Availability

Datasets generated and/or analyzed in this study are available from the corresponding author upon reasonable request.

## Abbreviations

CI: Confidence interval
COVID-19: Coronavirus disease 2019
FP: French Polynesia
IgG: Immunoglobulin class G
LI: Leeward Islands
MIA: Microsphere immunoassay
ROC: Receiver operating characteristic
SARS-CoV-2: Severe acute respiratory syndrome coronavirus 2
WHO: World Health Organization
WI: Winward Islands
