# Supplementary material for: SARS-CoV-2 seroprevalence and associated factors of infection before and after the Delta wave in French Polynesia: a cross-sectional study
Source: BMC Public Health. 2024 Feb 5;24:382. doi: 10.1186/s12889-024-17869-4 (PMC10840228; doi:10.1186/s12889-024-17869-4)
Supplement: Supplementary file 1 — Additional file 1: Supplementary Table 1. Sociodemographic characteristics of the study participants with available serological results for the detection of anti-SARS-CoV-2-N antibodies. [file 12889_2024_17869_MOESM1_ESM.docx]

Supplementary Table 1. Sociodemographic characteristics of the study participants with available serological results for the detection of anti-SARS-CoV-2-N antibodies

|  | | **Total**  **n (%)** | **Archipelago** | | | | | | | | | | | | | **p-value** |
| --- | --- | --- | --- | --- | --- | --- | --- | --- | --- | --- | --- | --- | --- | --- | --- | --- |
|  | |  | **Society (WIs)** | | **Society (LIs)** | | **Austral** | | **Marquesas** | | **Tuamotu** | | **Gambier** | | |  |
| ***Total, n (%)*** | | 1,120 (100) | 641 (57.2) | | 103 (9.2) | | 59 (5.3) | | 217 (19.4) | | 59 (5.3) | | 41 (3.6) | | |  |
| ***Males,*** ***n (%)*** | | 536 (47.9) | 318 (49.6) | | 42 (40.8) | | 31 (52.5) | | 102 (47.0) | | 22 (37.3) | | 21 (51.2) | | | 0.28 |
| ***Median age in years (IQR)*** | | 37 (28-50) | 37 (27-50) | | 41 (31-52) | | 36 (29-44) | | 39 (29-50) | | 38 (28-50) | | 39 (27-48) | | | 0.32 |
| ***Socio-cultural background, n (%)*** |  | | |  |  |  | |  | |  | |  | |  | -* | |
| Polynesian | | 841 (75.1) | 426 (66.5) | | 99 (96.2) | | 53 (89.8) | | 180 (82.9) | | 49 (83.0) | | 34 (82.9) | | |  |
| Caucasian | | 53 (4.7) | 31 (4.8) | | 2 (1.9) | | 0 (0) | | 13 (6.0) | | 5 (8.5) | | 2 (4.9) | | |  |
| Asian | | 10 (0.9) | 9 (1.4) | | 0 (0) | | 0 (0) | | 1 (0.5) | | 0 (0) | | 0 (0.0) | | |  |
| Mixed race | | 200 (17.9) | 162 (25.3) | | 2 (1.9) | | 4 (6.8) | | 22 (10.1) | | 5 (8.5) | | 5 (12.2) | | |  |
| Other | | 8 (0.7) | 5 (0.8) | | 0 (0) | | 2 (3.4) | | 1 (0.5) | | 0 (0) | | 0 (0) | | |  |
| Missing data | | 8 (0.7) | 8 (1.2) | | 0 (0) | | 0 (0) | | 0 (0) | | 0 (0) | | 0 (0) | | |  |
| ***Level of education, n (%)*** | |  |  | |  | |  | |  | |  | |  | | | <0.001** |
| Primary | | 164 (14.6) | 77 (12.0) | | 17 (16.5) | | 10 (16.9) | | 37 (17.1) | | 9 (15.2) | | 14 (34.2) | | |  |
| Secondary | | 276 (24.6) | 157 (24.5) | | 27 (26.2) | | 14 (23.7) | | 48 (22.1) | | 19 (32.2) | | 11 (26.8) | | |  |
| High School (or equivalent) | | 433 (38.7) | 235 (36.7) | | 50 (48.6) | | 29 (49.2) | | 87 (40.1) | | 24 (40.7) | | 8 (19.5) | | |  |
| University (or after) | | 246 (22.0) | 172 (26.8) | | 9 (8.7) | | 6 (10.2) | | 45 (20.7) | | 7 (11.9) | | 7 (17.1) | | |  |
| Missing data | | 1 (0.1) | 0 (0) | | 0 (0) | | 0 (0) | | 0 (0) | | 0 (0.0) | | 1 (2.4) | | |  |
| ***Marital status, n (%)*** | |  |  | |  | |  | |  | |  | |  | | | -* |
| Never married | | 292 (26.1) | 163 (25.4) | | 35 (34.0) | | 24 (40.7) | | 42 (19.4) | | 16 (27.1) | | 12 (29.2) | | |  |
| Cohabiting | | 336 (30.0) | 202 (31.5) | | 22 (21.3) | | 14 (23.7) | | 69 (31.8) | | 15 (25.4) | | 14 (34.2) | | |  |
| Married | | 420 (37.5) | 232 (36.2) | | 42 (40.8) | | 19 (32.2) | | 88 (40.5) | | 25 (42.4) | | 14 (34.2) | | |  |
| Separated, divorced, widowed | | 70 (6.2) | 43 (6.7) | | 4 (3.9) | | 2 (3.4) | | 17 (7.8) | | 3 (5.1) | | 1 (2.4) | | |  |
| Missing data | | 2 (0.2) | 1 (0.2) | | 0 (0.0) | | 0 (0.0) | | 1 (0.5) | | 0 (0.0) | | 0 (0.0) | | |  |

IQR: interquartile range; WIs: Windward Islands; LIs: Leeward Islands

* Fisher’s exact test not performed due to computational limitations; the conditions for the Chi2 test were not met (small numbers)

** Chi2 test performed without missing data
